# Statistics of the Medical Practitioners of Paris

**Published:** 1845-04

**Authors:** 


*Statistics of the Medical Practitioners of Paris.* — The following statistical table has just appeared in a work (Almanach Général de Médecine) published by M. Domange. The number of doctors in medicine and surgery, on the 1st of January, 1845, was 1,430; in 1833, there were 1,090; in 1836, 1,220; in 1839, 1,310; in 1841, 1,360; in 1843, 1,423. Of the 1,430, 1,323 are of the Faculty of Paris; 50 of that of Montpelier; 31 of that of Strasburgh; 26 from foreign universities, authorised to practise by *ordonnance royale;* 15 are doctors in surgery; 8 doctors in medicine and surgery; 10 are dentists; 14 oculists; 16 homœopathists; 4 magnetizers; 320 are members of the Legion of Honor (4 commanders, 50 officers, and 266 knights;) 42 do not practise, some from age and infirmities, others because exclusively occupied with scientific objects, and others, again, because they have taken to different careers. The mean number of deaths was, from 1834 to 1840, 20 per annum; and from 1840 to 1844, only 18. Finally, the number of officers of health is 168; pharmaciens, 326; and midwives, 450. To complete this table, I will add the number of inscriptions, or entries, taken on the 15th of November, 1844, at the Faculty of Medicine, Paris; they amounted to 800; in 1843, they were only 749. Of the 800 inscriptions, 251 were new students, viz: those commencing their medical studies, 199; those coming from the different preparatory schools, 43; those coming from the other faculties, 6.

*Med. News*, from *Med. Times.*

